# Mitigation of total body irradiation-induced mortality and hematopoietic injury of mice by a thrombopoietin mimetic (JNJ-26366821)

**DOI:** 10.1038/s41598-022-07426-7

**Published:** 2022-03-03

**Authors:** Vidya P. Kumar, Gregory P. Holmes-Hampton, Shukla Biswas, Sasha Stone, Neel Kamal Sharma, Bernadette Hritzo, Mary Guilfoyle, Gary Eichenbaum, Chandan Guha, Sanchita P. Ghosh

**Affiliations:** 1 Armed Forces Radiobiology Research Institute, Uniformed Services University of the Health Sciences, Bethesda, MD 20889 USA; 2 Office of the Chief Medical Officer, Johnson & Johnson, 410 George Street, New Brunswick, NJ 08901 USA; 3 Department of Radiation Oncology, Albert Einstein College of Medicine, Bronx, NY 10467 USA

**Keywords:** Drug discovery, Molecular biology, Biomarkers, Preclinical research

## Abstract

The threat of a nuclear attack has increased in recent years highlighting the benefit of developing additional therapies for the treatment of victims suffering from Acute Radiation Syndrome (ARS). In this work, we evaluated the impact of a PEGylated thrombopoietin mimetic peptide, JNJ-26366821, on the mortality and hematopoietic effects associated with ARS in mice exposed to lethal doses of total body irradiation (TBI). JNJ-26366821 was efficacious as a mitigator of mortality and thrombocytopenia associated with ARS in both CD2F1 and C57BL/6 mice exposed to TBI from a cobalt-60 gamma-ray source. Single administration of doses ranging from 0.3 to 1 mg/kg, given 4, 8, 12 or 24 h post-TBI (LD70 dose) increased survival by 30–90% as compared to saline control treatment. At the conclusion of the 30-day study, significant increases in bone marrow colony forming units and megakaryocytes were observed in animals administered JNJ-26366821 compared to those administered saline. In addition, enhanced recovery of FLT3-L levels was observed in JNJ-26366821-treated animals. Probit analysis of survival in the JNJ-26366821- and saline-treated cohorts revealed a dose reduction factor of 1.113 and significant increases in survival for up to 6 months following irradiation. These results support the potential use of JNJ-26366821 as a medical countermeasure for treatment of acute TBI exposure in case of a radiological/nuclear event when administered from 4 to 24 h post-TBI.

## Introduction

Risk of radiation exposure due to terrorist attacks or nuclear accidents is increasing and it is critical to have multiple radiation countermeasures readily available. The extent of injury resulting from exposure to ionizing radiation is dependent on the extent of exposure, with the hematopoietic system being the most susceptible^1^. Development of radiation countermeasures for treatment of Hematopoietic Acute Radiation Syndrome (H-ARS) has focused on drugs that promote hematopoietic recovery and more specifically, agents that stimulate production of white cells and platelets following Total Body Irradiation (TBI)^2,3^.

To date, the FDA has approved 4 radiation countermeasures for the mitigation of H-ARS, each serving to stimulate white cells or platelets: Neupogen^4^, Neulasta^5^, Leukine^6^, and Nplate^7^. Neupogen (filgrastim) and Neulasta (PEGylated filgrastim) were approved in 2015 and are both recombinant forms of the cytokine, Granulocyte-colony stimulating factor (G-CSF). Neupogen (filgrastim) requires daily dosing until neutrophil counts are above 1000/ml for 3 consecutive readings and Neulasta (PEGylated filgrastim) requires two doses administered a week apart. Both Neupogen and Neulasta mitigate H-ARS by facilitating bone marrow recovery (especially neutrophil recovery) which aids in maintaining immune function and capacity for fighting infections. Leukine was approved in 2018 and is a recombinant form of the Granulocyte–macrophage colony-stimulating factor (GM-CSF) cytokine. Leukine promotes proliferation of various cell types including macrophages and eosinophils and requires daily dosing. Nplate (romiplostim) is a thrombopoietin (TPO) mimetic IgG conjugated peptide with no sequence homology to endogenous TPO that stimulates platelet production. It was approved in 2021 to treat ARS based on data showing enhanced survival and platelet recovery in pre-clinical models of ARS^8,9^.

Romiplostim is one of the four TPO mimetics that have been developed and approved for thrombocytopenia-related indications^10,11^ and is the only one approved for ARS treatment. Interest in evaluating TPO mimetics for treatment of ARS was spurred by findings that recombinant human TPO enhanced survival and platelet recovery^12^. However, generation of autoantibodies to endogenous TPO was reported in some patients treated with recombinant human TPO^13^, highlighting the need for TPO mimetics. JNJ-26366821 (TPOm) is a novel TPO mimetic that, like the approved TPO mimetics, has no sequence homology to endogenous TPO, mitigating the risk for generation of autoantibodies to endogenous TPO and subsequent thrombocytopenia. Like romiplostim, JNJ-26366821 is a synthetic peptide that can be administered subcutaneously or intravenously, whereas the other TPO mimetics are orally administered small molecules. JNJ-26366821 has a similar but distinct peptide sequence to romiplostim and is PEGylated to extend its half-life whereas romiplostim is IgG conjugated^14^. JNJ-26366821 has been evaluated in chronic toxicity studies^15,16^, in a Phase 1 study to determine safety and tolerability in healthy volunteers^16^ and a Phase 1b study in cancer patients (internal results of Janssen). Given the unique properties of JNJ-26366821 and the need for multiple radiation countermeasures, it was of interest to evaluate the efficacy and safety of JNJ-26366821 in pre-clinical models of H-ARS.

Here, we present the effects of JNJ-26366821 on survival and hematopoietic recovery in mouse models of ARS when administered between four and twenty-four hours post-TBI. The results support future development of JNJ-26366821 as radiation mitigator.

## Results

### JNJ-26366821 administered 4–24 h post-TBI increases survival

**Figure 1:**
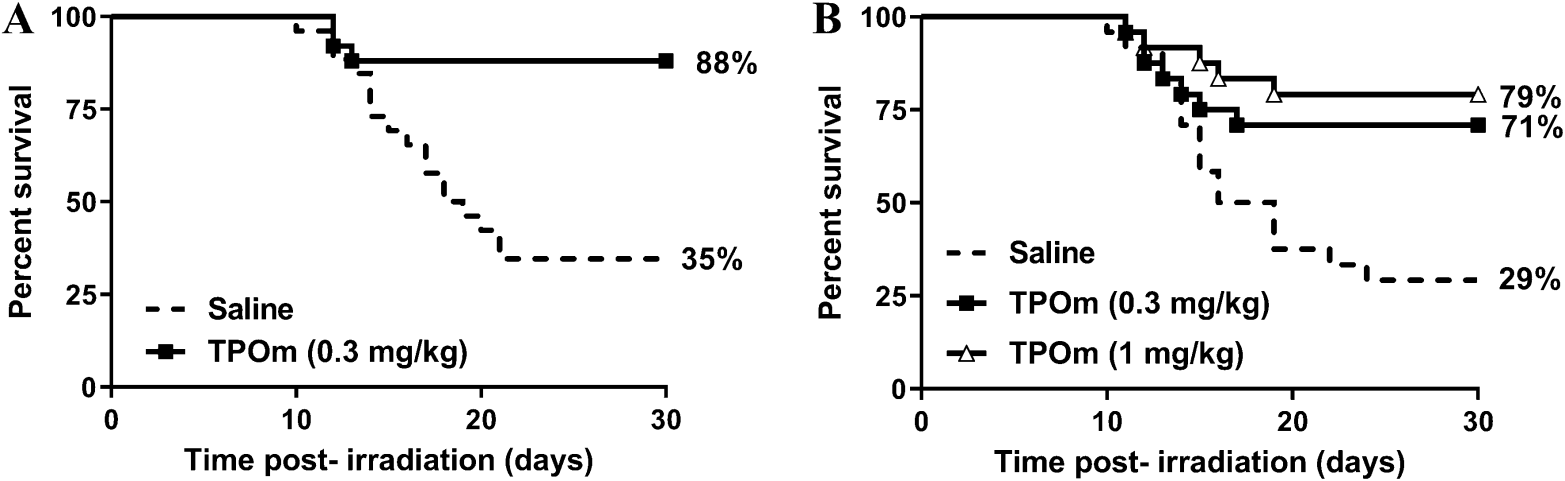
Survival of CD2F1 mice following total body irradiation with 9.35 Gy. A single dose of JNJ-26366821 at 0.3 mg/kg (■) or saline as a vehicle (dashed line) was administered 4 h post-TBI (**A**) (Log-rank test *p* = 0.0002) , a single dose of JNJ-26366821 either at 0.3 mg/kg (■) or at 1.0 mg/kg (Δ) or saline as a vehicle (dashed line) was administered 24 h post-TBI (**B**) (Log-rank test Saline vs. JNJ-26366821 (0.3 mg/kg)* p* = 0.0122, Saline vs. JNJ26366821 (1.0 mg/kg) *p* = 0.0008, JNJ-26366821 (0.3 mg/kg) vs. JNJ-26366821 (1.0 mg/kg) *p* = 0.4787. Kaplan–Meier survival curves were plotted using GraphPad software; n = 24 mice per group and trend in survival is compared between vehicle and drug-treated groups. Figures were plotted using GraphPad Prism 9 software V9.0.2; www.graphpad.com.

Prior to TBI studies, TPOm (single dose of 3 mg/kg) was tested for safety and tolerability in CD2F1 male mice and was found to be safe at this dose with no abnormal clinical signs of toxicity and the absence of pathological findings on gross necropsy. JNJ-26366821 (0.3 mg/kg) or saline were administered 4 h post-TBI at 9.35 Gy (LD70/30) and monitored for survival. At the conclusion of the study, 88% of animals administered JNJ-26366821 survived whereas 35% of animals administered saline survived (Fig. 1A). Comparing the survival curves using Log-rank test demonstrated a significant difference (*p* = 0.0002) between the saline and JNJ-26366821 curves; comparing the survival percentage in both groups at day 30 with a Fisher’s exact test (88% vs 35%) was also statistically significant (*p* = 0.0001). Additional animals were administered either JNJ-26366821 (0.3 mg/kg or 1.0 mg/kg) or saline 24 h post-TBI; survival in animals administered 1.0 mg/kg JNJ-2636682, 0.3 mg/kg JNJ-26366821, and saline was 79%, 71%, and 29%, respectively. (Fig. 1B).

Log-rank test comparison of the survival curves demonstrated a significant increase in survival in both JNJ-26366821-treated groups compared to the saline-treated group (1.0 mg/kg JNJ-26366821 vs saline, *p* = 0.0008 and 0.3 mg/kg JNJ-26366821 vs saline, *p* = 0.0122); survival in JNJ-26366821 groups was not significantly different (*p* = 0.4787). Comparison of survival outcome at day 30 (Fisher’s exact test), demonstrated significantly increased survival in both JNJ-26366821-treated groups over saline (1.0 mg/kg JNJ-26366821 (79%) vs saline (29%), *p *= 0.0012 and 0.3 mg/kg JNJ-26366821 (71%) vs saline (29%), *p* = 0.0087); and no significant difference in survival outcome between the two JNJ-26366821-treated groups (79% vs 71%, *p* = 0.7400). Additionally, survival following multiple doses of JNJ-26366821 (a 1, 2, or 3 dose regimen administered once daily) was assessed however, no additional survival benefit was observed in animals administered more than a single dose of JNJ-26366821 (Supplemental Fig. 1).

### JNJ-26366821 dose-dependently enhances survival post-TBI

**Figure 2:**
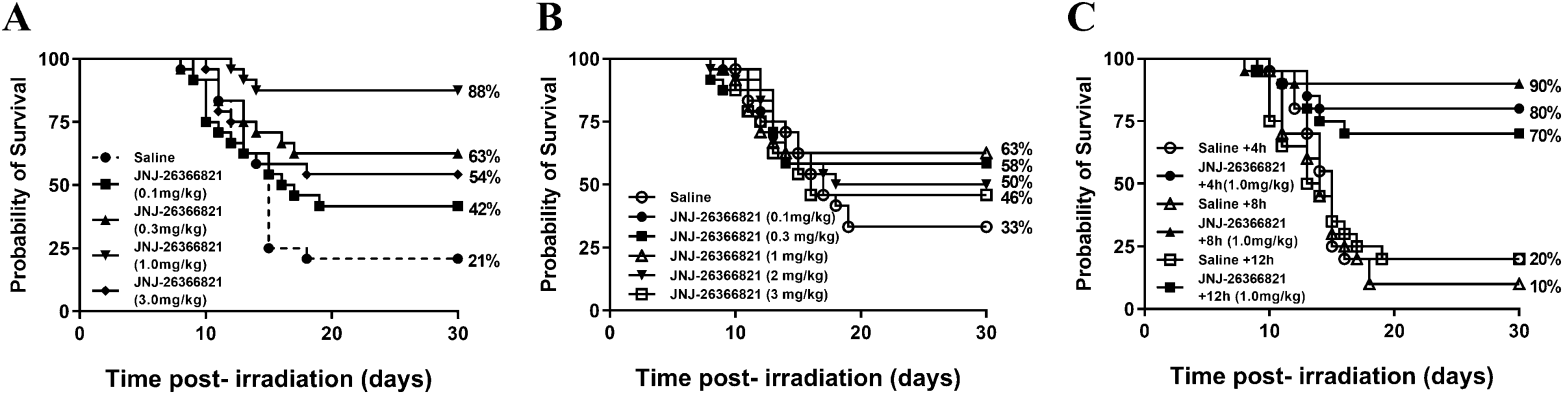
Time and dose optimization of JNJ-26366821 administration. (**A**) Optimization of JNJ-26366821 dose to improve survival of lethally irradiated CD2F1 mice when administered SC 4 h after 9.75 Gy TBI (n = 24/group). Survival curves shown here are saline as the vehicle (●) and JNJ-26366821 at doses of 0.1 mg/kg (■), 0.3 mg/kg (▲), 1.0 mg/kg (▼), and 3.0 mg/kg (♦), Percent survival on day 30 post-TBI is shown at the end of each curve. Kaplan–Meier survival curves were plotted using GraphPad software; n = 24 mice per group and trend in survival is compared between vehicle and drug-treated groups (Log-rank test *p* = 0.0001–0.2515). (**B**) Optimization of JNJ-26366821 dose to improve survival of lethally irradiated CD2F1 mice when administered SC 24 h after 9.35 Gy TBI (n = 24/group). Survival curves shown here are saline as the vehicle (○) and JNJ-26366821 doses 0.1 mg/kg (●), 0.3 mg/kg (■), 1.0 mg/kg (Δ), 2.0 mg/kg (▼) and 3.0 mg/kg (□). Percent survival on day 30 post-TBI is shown at the end of each curve. Kaplan–Meier survival curves were plotted using GraphPad software; n = 24 mice per group and trend in survival is compared between vehicle and drug-treated groups. (**C**) Effect of TPOm administration SC at 4, 8 and 12 h post-TBI (9.75 Gy, n = 24/group) in CD2F1 mice. Survival curves shown here are saline as the vehicle at 4 h (○), 8 h (Δ), 12 h (□) and JNJ-26366821 at 4 h (●), 8 h (▲) and 12 h (■) post-TBI. Percent survival values on day 30 post-TBI are shown at the end of each curve. Kaplan–Meier survival curves were plotted using GraphPad software; n = 20 mice per group and trend in survival is compared between vehicle and drug-treated groups (Log-rank test *p* = 0.0001–0.0019). Figures were plotted using GraphPad Prism 9 software V9.0.2; www.graphpad.com.

Male CD2F1 mice were irradiated at 9.75 Gy and administered saline or JNJ-26366821 at various concentrations (0.1, 0.3, 1.0, and 3.0 mg/kg). The highest survival was observed in animals administered 1.0 mg/kg JNJ-26366821 (88%), followed by 63%, 54%, 42%, and 21% survival in animals administered 0.3 mg/kg JNJ-26366821, 3.0 mg/kg JNJ-26366821, 0.1 mg/kg JNJ-26366821, and saline, respectively (Fig. 2A). Log-rank comparison of the survival curves show statistical differences between treatment with 0.3 mg/kg and 1.0 mg/kg JNJ-26366821 (*p* = 0.0106 and *p* < 0.0001, respectively); survival in animals treated with 0.1 mg/kg and 3.0 mg/kg JNJ-26366821 was not statistically different than that in saline-treated animals (Log-rank *p* = 0.2515 and *p* = 0.0734, respectively). When comparing the survival curves (Log-rank test) for the various JNJ-26366821-treated groups, survival in the groups that received 0.1 mg/kg and 1.0 mg/kg were statistically different (*p* = 0.0009), whereas survival curves for animals administered 0.1 and 0.3 or 0.1 and 3.0 mg/kg were not statistically different (*p* = 0.1599 and *p *= 0.3606, respectively). Survival curves for animals that received 0.3 and 1.0 mg/kg were statistically different (*p* = 0.0451) whereas survival curves from animals receiving 0.3 and 3.0 mg/kg were not (*p* = 0.5674). Finally, survival curves for animals administered 1.0 mg/kg were statistically different from those administered 3.0 mg/kg (*p* = 0.0096). All log-rank statistical analyses are summarized in Table 1 (top set of values). In total, the group that was administered 1.0 mg/kg JNJ-26366821 was the only group was statistically different from all other groups.

**Table 1:** Comparison of Log-Rank (top) and Fisher’s exact test (bottom) statistical analyses for Fig. 2A.

Group	Saline	0.1 mg/kg JNJ-26366821	0.3 mg/kg JNJ-26366821	1.0 mg/kg JNJ26366821	3.0 mg/kg JNJ-26366821
Saline	–	*p* = 0.2515	*p* = 0.0106	*p* < 0.0001	*p* = 0.0734
0.1 mg/kg JNJ-26366821	*p* = 0.2124	–	*p* = 0.1599	*p* = 0.0009	*p* = 0.3606
0.3 mg/kg JNJ-26366821	*p* = 0.0077	*p* = 0.2476	–	*p* = 0.0451	*p* = 0.5674
1.0 mg/kg JNJ26366821	*p* < 0.0001	*p* = 0.0020	*p* = 0.0933	–	*p* = 0.0096
3.0 mg/kg JNJ-26366821	*p* = 0.0355	*p* = 0.5639	*p* = 0.7702	*p* = 0.0243	–

Above the diagonal of non-comparison where groups would be compared with themselves are significance values for log-rank analysis of the survival curves. Below the diagonal are significance values for Fisher’s exact test conducted based on surviving proportions on day 30.

Comparing the 30-day survival (Fisher’s exact test) of animals administered saline or JNJ-26366821, survival was significantly increased in animals administered 0.3 mg/kg, 1.0 mg/kg, and 3.0 mg/kg JNJ-26366821 over the saline control (*p* = 0.0077, *p* < 0.0001, and *p* = 0.0355 respectively) no statistical difference was observed in the survival of animals administered 0.1 mg/kg JNJ-26366821 as compared to saline (*p* = 0.2124). Comparison of 30-day survival (Fisher’s exact test) in the various JNJ-26366821 dose groups showed survival for animals administered 0.1 mg/kg was lower than animals administered 1.0 mg/kg JNJ-26366821 (*p* = 0.0020), whereas survival for animals administered 0.1 and 0.3 or 0.1 and 3.0 mg/kg JNJ-26366821 was not statistically different from one another (*p* = 0.2476 and *p* = 0.5639, respectively). Survival for animals that received 0.3 and 1.0 mg/kg or 0.3 and 3.0 mg/kg were not statistically different from one another (*p* = 0.0933 and *p* = 0.7702, respectively). Finally, survival curves for animals administered 1.0 mg/kg were statistically different from those administered 3.0 mg/kg (*p* = 0.0243).

Similar analyses were conducted for male CD2F1 mice administered saline or JNJ-26366821 (0.1, 0.3, 1.0, 2.0, and 3.0 mg/kg) 24 h after TBI at 9.35 Gy. The highest survival was observed in animals administered 1.0 mg/kg JNJ-26366821 (63%), followed 58%, 50%, 46%, and 33% survival in animals administered 0.1 and 0.3 mg/kg JNJ-26366821, 2.0 mg/kg JNJ-26366821, 3.0 mg/kg JNJ-26366821 and saline, respectively (Fig. 2B). These data indicated the optimal JNJ-26366821 dose was 1.0 mg/kg however the modest separation in survival between saline and the various doses of JNJ-26366821 did not result in statistical significance. Comparing the groups administered saline and 1.0 mg/kg JNJ-26366821 (lowest and highest survival, respectively), the Log-rank test (*p* = 0.1402) and Fisher’s exact test at day 30 (*p* = 0.0820) were the closest to statistical significance.

### JNJ-26366821 enhances survival up to 24 h post-TBI

To evaluate the impact of time of administration at the maximally effective dose of 1.0 mg/kg JNJ-26366821, male CD2F1 mice were irradiated at 9.75 Gy and administered JNJ-26366821 or saline at either 4, 8, or 12 (Fig. 2C) or 24 (Fig. 1B) hours post-TBI. Survival in animals administered JNJ-26366821 or saline 4 h post-TBI was 80% and 20% respectively; a log-rank test comparing the curves showed significant difference (*p* = 0.0006) and a Fisher’s exact test comparing survival at day 30 demonstrated a significant increase in survival in animals administered JNJ-26366821 (*p* = 0.0004). For animals administered JNJ-26366821 or saline 8 h post-TBI, survival was 90% and 10% respectively; log-rank comparison of the curves revealed significant difference (*p* < 0.0001) and a Fisher’s exact test comparing survival at day 30 demonstrated a significant increase in survival in animals administered JNJ-26366821 (*p* < 0.0001). Survival in animals administered JNJ-26366821 or saline 12 h post-TBI was 70% and 20% respectively; the curves were significantly different as shown by log-rank test (*p* = 0.0019) and a Fisher’s exact test comparing survival at day 30 showed a significant increase in survival in animals administered JNJ-26366821 (*p* = 0.0036). Comparison of survival in the groups administered JNJ-26366821 at different time points did not reveal statistically significant differences, + 4 h vs + 8 h (Log-rank test: *p* = 0.6392, 30 day Fisher’s exact test: *p* = 0.6614), + 4 h vs + 12 h (Log-rank test: *p* = 0.5074, 30 day Fisher’s exact test: *p* = 0.7164), and + 8 h vs + 12 h (Log-rank test: *p* = 0.1404, 30 day Fisher’s exact test: *p* = 0.2351). Similarly, no significant differences were observed amongst the groups administered saline.

### JNJ-26366821 administered 24 h post-TBI enhances recovery of neutrophils, platelets, and bone marrow progenitor cells

**Figure 3:**
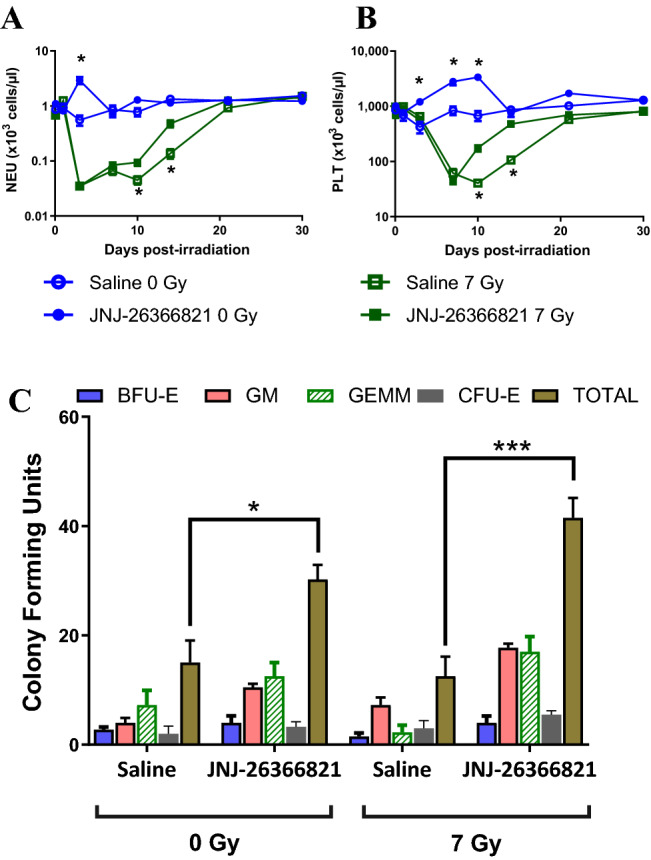
Recovery of peripheral blood cells (neutrophils (NEU) and platelets (PLT)) and bone marrow colony forming units. (**A**) & (**B**) Blood cell counts for days 0 to 30: Saline or JNJ-26366821 at 1 mg/kg was administered 24 h post-irradiation. Day 0 represents 2 h post-irradiation. Non-irradiated mice treated with saline (○) and JNJ-26366821 (●) and irradiated (7 Gy) mice treated with saline (□) and JNJ-26366821 (■). Data represented are mean ± standard error of the mean (SEM) for n = 10 mice. Significant difference (*p* < 0.001–0.0125) between TPOm treated and saline treated irradiated groups by ANOVA is indicated with an asterisk (*). Some data points in the figure do not have error bars that are visible because they are smaller than symbols. (**C**) Bone Marrow colony forming units for animals administered Saline or JNJ-26366821 (1 mg/kg) both 24 h after irradiation (7 Gy) and in non-irradiated animals (0 Gy) Significant difference *p = 0.04, ***p = 0.0010. Figures were plotted using GraphPad Prism 9 software V9.0.2; www.graphpad.com.

A 1.0 mg/kg dose of JNJ-26366821 or saline was administered to CD2F1 mice 24 h post-TBI at a nonlethal dose (7 Gy). Blood was collected at various time points in the 30-day study; blood was also collected at the same time points from an additional cohort of animals that were not exposed to irradiation but received 1.0 mg/kg JNJ-26366821 or saline. Amongst the non-irradiated animals (blue traces, Fig. 3A), JNJ-26366821 administration was sufficient to increase neutrophil counts on day 3 (2.931 ± 1.376 × 10^3^cells/μl of blood for animals administered JNJ-26366821 vs. 0.553 ± 0.348 × 10^3^cells/μl of blood for animals administered saline, *p* = 0.0001). Amongst irradiated animals (green traces, Fig. 3A), neutrophil counts were elevated in animals administered JNJ-26366821 compared to those administered saline 24 h post-TBI on days 10 and 14 (day 10: 0.094 ± 0.040 × 10^3^cells/μl of blood for animals administered JNJ-26366821 vs. 0.045 ± 0.024 × 10^3^cells/μl of blood for animals administered saline, *p* = 0.0040 and day14: 0.477 ± 0.223 × 10^3^cells/μl of blood for animals administered JNJ-26366821 vs. 0.137 ± 0.085 × 10^3^cells/μl of blood for animals administered saline, *p* = 0.0003).

In the non-irradiated animals (blue traces, Fig. 3B), JNJ-26366821 elevated platelet counts compared to saline on days 3, 7, and 10 (day 3: 1201 ± 269 × 10^3^cells/μl vs 422 ± 300 × 10^3^cells/μl, *p* < 0.0001, day 7: 1201 ± 269 × 10^3^cells/μl vs 422 ± 300 × 10^3^cells/μl, *p* = 0.0002, and day 10: 1201 ± 269 × 10^3^cells/μl vs 422 ± 300 × 10^3^cells/μl, *p* < 0.0001). In the irradiated animals (green traces, Fig. 3B), JNJ-26366821 accelerated recovery of platelet counts compared to saline on days 10 and 14 (day 10: 176 ± 76 × 10^3^cells/μl vs 41 ± 10 × 10^3^cells/μl, *p* < 0.0001 and day 14: 478 ± 113 × 10^3^cells/μl vs 106 ± 40 × 10^3^cells/μl, *p* < 0.0001). Taken together, these results demonstrate an accelerated recovery of these cell types in animals administered 1.0 mg/kg JNJ-26366821 24 h post-TBI. Additional cell types were also analyzed including white blood cells, monocytes, and lymphocytes (Supplemental Figs. 2A–C). These measurements showed these three cell types were elevated in non-irradiated animals administered JNJ-26366821 compared to those administered saline on day 3 and all three cell types were elevated in irradiated animals administered JNJ-26366821 compared to those administered saline on day 14. Red blood cells were also analyzed (Supplemental Fig. 2D) however there were no statistically significant changes on any days.

At the conclusion of the 30-day study, bone marrow was collected from the same animals and assayed for colony forming units (Fig. 3C). Total bone marrow colony forming units (CFU-GM, CFU-GEMM, CFU-E, and BFU-E) in the non-irradiated animals were elevated in animals administered JNJ-26366821 (1.0 mg/kg) compared to those administered saline (30.3 ± 5.3 colonies vs 15.0 ± 8.2 colonies, *p* = 0.0440). Similarly, bone marrow colony forming units in the irradiated animals were elevated in animals administered JNJ-26366821 compared to those administered saline (41.5 ± 7.3 colonies vs 12.5 ± 7.2 colonies, *p* = 0.0010).

### JNJ-26366821 enhances survival and hematopoietic recovery in an additional mouse strain

**Figure 4:**
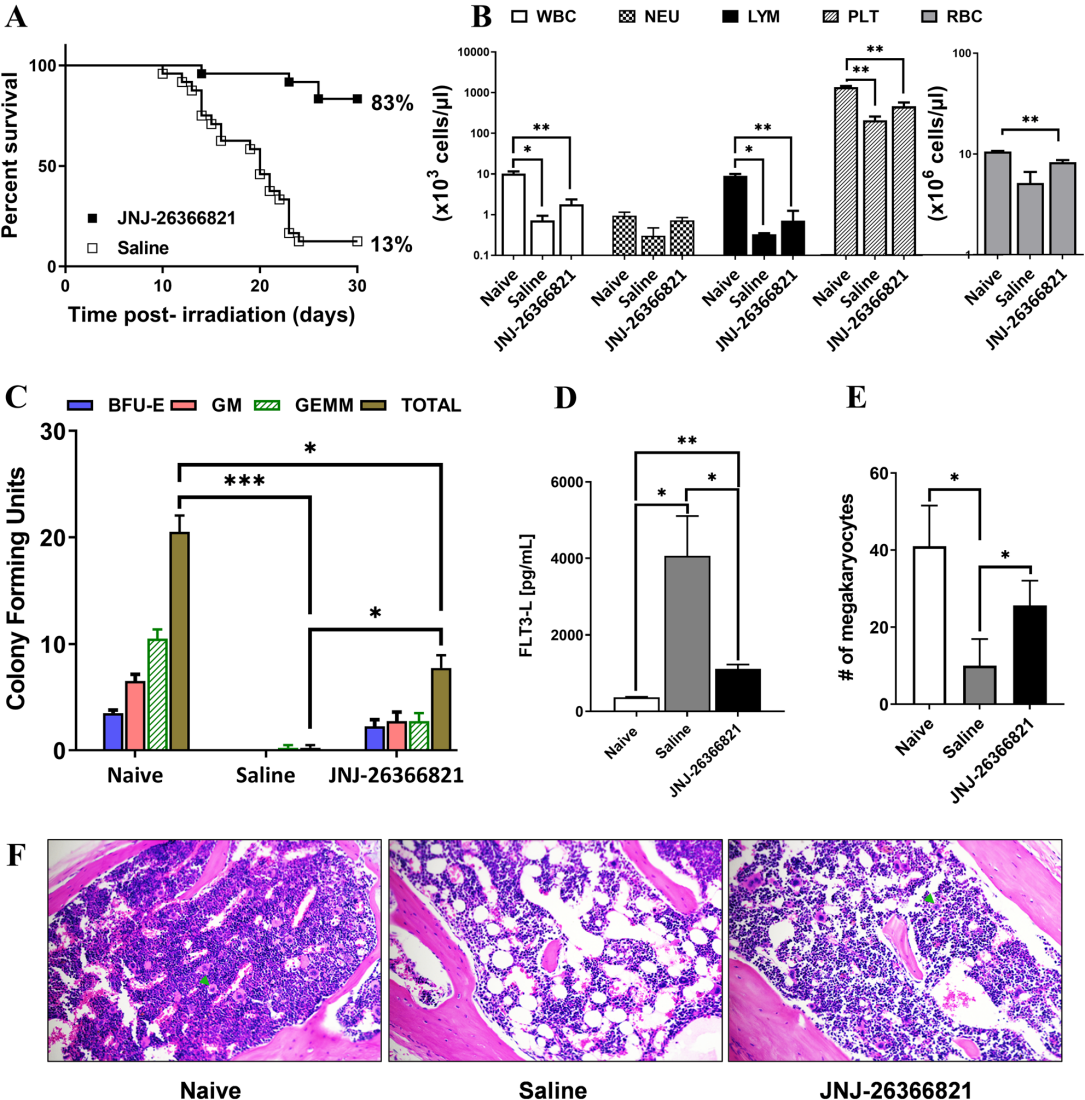
(**A**) Survival in C57BL/6 male mice following 8.0 Gy total body irradiation (TBI). A single dose of JNJ-26366821 at 1 mg/kg (■) or saline as a vehicle (□) was administered 24 h post-TBI to C57BL/6 male mice. Kaplan–Meier survival curves were plotted using GraphPad software; n = 24 mice per group and trend in survival is compared between vehicle and drug-treated groups (Log-rank test *p* < 0.0001). Percent survival on day 30 post-TBI is shown at the end of each curve. (**B**–**E**) The non-irradiated naïve group was compared with the irradiated groups (saline and JNJ-26366821). (**B**) Peripheral blood cell counts on day 30 post-TBI (8 Gy). White blood cells (WBC), neutrophils (NEU), lymphocytes (LYM), red blood cells (RBC) and platelets (PLT) were measured. (**C**) Recovery of femoral bone marrow was estimated by Colony forming Unit assay. On day 30 post-TBI (8 Gy), the JNJ-26366821 group had significant recovery (*p* = 0.01) compared to saline group. (**D**) Serum levels of FLT3-L on day 30 post-TBI (8 Gy) were measured by ELISA showing recovery by JNJ-26366821 treatment. Data represented are mean ± standard error of the mean (SEM) for n = 3 mice. (**E**) Recovery of cellularity of sternal bone marrow was determined on day 30 post-TBI (8 Gy). The number of megakaryocytes were counted in all three groups. The JNJ-26366821 group showed significant recovery (*p* < 0.05) compared to saline group. Significant difference (*p* < 0.001–0.05) by t-test is indicated with an asterisk (*). (**F**) Representative sternal bone marrow sections are shown for naïve, irradiated saline and JNJ-26366821 groups. Green arrows indicate megakaryocytes. Figures were plotted using GraphPad Prism 9 software V9.0.2; www.graphpad.com.

The increased survival efficacy of JNJ-26366821 treatment in irradiated CD2F1 mice was confirmed using the C57BL/6 strain. C57BL/6 male mice were exposed to 8.0 Gy (TBI) and administered 1.0 mg/kg JNJ-26366821 24 h post-TBI (Fig. 4A). Survival in animals administered JNJ-26366821 was 83% whereas survival in the saline-treated animals was 13%; a log-rank test confirmed the survival curves were statistically different from one another (*p* < 0.0001) and a Fisher’s exact test revealed survival was statistically higher in the animals administered JNJ-26366821 (*p* < 0.0001).

At the conclusion of the 30-day study, blood, femoral bone marrow, and sterna were collected from C57BL/6 animals in the survival study (Fig. 4A) which included those administered either saline or 1.0 mg/kg JNJ-26366821 24 h post-TBI at 8.0 Gy as well as age matched, non-irradiated naïve animals. Complete blood cell counts were assessed in whole blood (Fig. 4B). White blood cells counts were highest in naïve animals (10.2 ± 2.3 × 10^3^ cells/μl) and were significantly higher in the naïve group than in either irradiated group (saline 0.72 ± 0.38 × 10^3^ cells/μl, *p* = 0.0164 and JNJ-26366821 1.8 ± 1.0 × 10^3^ cells/μl, *p* = 0.0042); there was no statistical difference between the counts in the irradiated groups (*p* = 0.1553). Neutrophil counts were also highest in naïve animals (0.95 ± 0.36 × 10^3^ cells/μl) and higher in the naïve group than either irradiated group, although not significantly (saline 0.303 ± 0.291 × 10^3^ cells/μL, *p* = 0.0707 and JNJ-26366821 0.723 ± 0.224 × 10^3^ cells/μl, *p* = 0.3955). Additionally, the counts in the irradiated groups were not statistically different from one another (*p* = 0.1188). Lymphocytes counts were highest in naïve animals (9.0 ± 1.8 × 10^3^ cells/μl) and significantly higher in the naïve group than either irradiated group (saline 0.33 ± 0.04 × 10^3^ cells/μl, *p* = 0.0148 and JNJ-26366821 0.72 ± 0.22 × 10^3^ cells/μl, *p* = 0.0023); the counts in the irradiated groups were not statistically different from one another (*p* = 0.5157). For red blood cells, counts were not statistically higher in naïve animals (10.6 ± 0.3 × 10^6^ cells/μl) as compared to animals administered saline (5.2 ± 2.5 × 10^6^ cells/μl, *p* = 0.0652) however naïve counts were higher than counts in animals administered JNJ-26366821 (8.3 ± 0.7 × 10^6^ cells/μl, *p* = 0.0058); no significant difference was observed between counts in the irradiated groups (*p* = 0.1080). Finally, the highest platelet counts were observed in naïve animals (1364 ± 170 × 10^3^ cells/μl) and counts in naïve group were significantly higher than counts in either irradiated group (saline 209 ± 86 × 10^3^ cells/μl, *p* = 0.0020 and JNJ-26366821 463 ± 189 × 10^3^ cells/μl, *p* = 0.0036); the counts in irradiated groups were not statistically different from one another (*p* = 0.1011). For all cell type counts, the prevailing trend was naïve > JNJ-26366821 > saline.

Analysis of the total colony forming units (CFU-GM, CFU-GEMM, CFU-E, and BFU-E) in the bone marrow of these animals (Fig. 4C) demonstrated highest counts in naïve animals (20.5 ± 3.1) that were significantly elevated relative to animals administered saline (0.25 ± 0.50, *p* = 0.0006) or JNJ-26366821 (7.8 ± 2.6, *p* = 0.0150). CFU counts were also higher in animals administered JNJ-26366821 over those in animals administered saline (*p* = 0.0109). Similarly, circulating FLT3-L, as assessed by ELISA in serum (Fig. 4D), was significantly higher in the saline (4065 ± 1800 pg/ml) group as compared to both the naïve group (368 ± 21 pg/ml, *p* = 0.0236) and JNJ-26366821 group (1114 ± 191 pg/ml, *p* = 0.0476); FLT3-L levels were elevated in animals administered JNJ-26366821 as compared to naïve levels (*p* = 0.0025). Finally, megakaryocytes counts (Fig. 4E) in H&E stained sternum (Fig. 4F) were highest in naïve animals (41.0 ± 10.6) and were significantly elevated relative to those in animals administered saline (10.0 ± 6.9, *p* = 0.0132) but not those administered JNJ-26366821 (25.7 ± 6.4, *p* = 0.0986). Additionally, counts were higher in animals administered JNJ-26366821 than animals administered saline (*p* = 0.0454).

### JNJ-26366821 has a favorable dose reduction factor and enhances long term survival of C57BL/6 male mice

**Figure 5:**
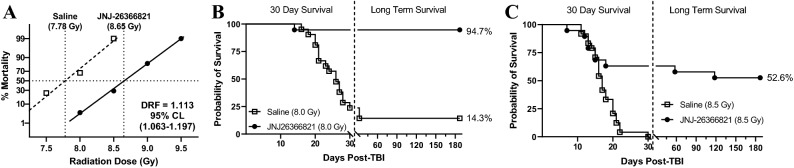
Dose reduction factor and long term survival in C57BL/6 male mice. **(A)** DRF analysis of animals administered JNJ-26366821 at 1 mg/kg (■) or saline as a vehicle (□) 24 h post-TBI. Probit analysis was performed using IBM SPSS statistical analysis software and estimated survival was plotted as dashed lines (Saline) or Solid lines (TPOm). LD50/30 values were determined to be 7.78 Gy and 8.65 Gy for Saline and JNJ-26366821, respectively yielding a DRF value of 1.113. N = 24 mice per group. **(B)** 30 day and 6 month survival data for animals administered Saline (□) or JNJ-26366821 (■) 24 h after irradiation at 8.0 Gy. Kaplan–Meier survival curves were plotted using GraphPad software; n = 19–22 mice per group and the trend in survival was compared between vehicle and drug-treated groups (Log-rank test *p* < 0.0001). **(C)** 30 day and 6 month survival data for animals administered Saline (□) or JNJ-26366821 (■) 24 h after irradiation at 8.5 Gy. Kaplan–Meier survival curves were plotted using GraphPad software; n = 19–24 mice per group and trend in survival is compared between vehicle and drug-treated groups (Log-rank test *p* = 0.0003). Figures were plotted using GraphPad Prism 9 software V9.0.2; www.graphpad.com.

Male C57BL/6 mice were administered saline or JNJ-26366821 (1.0 mg/kg) 24 h after TBI at various radiation doses to determine the dose reduction factor (Fig. 5A). By plotting survival for the various radiation dose groups treated with either saline or JNJ-26366821, the dose of radiation correlating to 50% lethality over 30 days (LD50/30) was determined by probit analysis. For animals administered saline, the LD50/30 dose was 7.78 Gy (95% confidence interval: 7.65–7.90 Gy) and for animals administered JNJ-26366821 the LD50/30 dose was 8.65 Gy (95% confidence interval: 8.52–8.79 Gy). In conjunction, these values indicated a dose reduction factor of 1.113 (95% confidence interval: 1.063–1.197). From this study, the survival of animals irradiated at 8.0 and 8.5 Gy was monitored for 6 months (Fig. 5B and C, respectively). Analysis of the survival curves for animals irradiated at 8.0 Gy indicated the curves were significantly different between saline and JNJ-2636682-treated groups as determined by log-rank test (*p* < 0.0001) and that survival was significantly higher at 6 months in animals administered JNJ-26366821 compared to those administered saline (94.7% vs 14.3%, *p* < 0.0001). Analysis of the survival curves for animals irradiated at 8.5 Gy indicated the curves were significantly different between saline and JNJ-2636682-treated groups as determined by log-rank test (*p* = 0.0003) and that survival was significantly higher at 6 months in animals administered JNJ-26366821 compared to those administered saline (52.6% vs 0%, *p* < 0.0001).

## Discussion

Based on its favorable safety and tolerability profile in animals^15^ and humans^16^, we evaluated the potential of JNJ-26366821 to enhance survival and hematopoietic recovery in pre-clinical mouse models of H-ARS. A single dose of TPOm administered either 4, 8, 12, or 24 h post-TBI enhanced 30-day survival significantly. In contrast, a single dose of recombinant human TPO (rhTPO) administered either 2 h before or 2 h after TBI increased 30-day survival however, no efficacy was observed when administration was delayed to 24 h post-TBI^17^. In addition, JNJ-26366821 is effective in multiple strains of mice, suggesting its use in diverse populations. Multiple dosing with JNJ-26366821 in our irradiated mouse model did not show additional benefit over a single administration 24 h post-TBI.

It is known that radiation-induced neutropenia and thrombocytopenia contribute significantly to lethality as a result of damage to the progenitor cells in the bone marrow^18–20^. To test the hypothesis that JNJ-26366821 increases thrombopoiesis in irradiated animals, which may partly contribute to survival benefit, we analyzed circulation peripheral blood cells in non-lethally irradiated (7 Gy) mice at 8 time points throughout the 30-day period following irradiation. This dose of radiation in the CD2F1 strain has been shown to induce severe bone marrow myelosuppression without associated mortality^21^. Since blood was collected through submandibular vein (only 20 µl) without sacrificing the animals, this model allows for multiple sampling times with surviving irradiated controls. The increase in circulating platelets by JNJ-26366821 supports our hypothesis that improvement in survival after JNJ-26366821 administration may be partly due to the thrombopoietic nature of the drug. The increased neutrophil counts observed after JNJ-26366821 administration may be due to stem cell mobilization into peripheral blood. Since JNJ-26366821 is shown to function by interaction through cMpl receptor, the drug’s ability to rescue mice from radiation-induced lethality could be attributed to stimulation of bone marrow to promote faster recovery of progenitor cells^22–24^. In addition, the effect of JNJ-26366821 in protection of vasculature integrity may have also contributed to the survival benefit^25^.

There are data showing that patients who switched between romiplostim or the FDA approved TPO mimetic eltrombopag, either due to lack of efficacy or for other reasons such as adverse side effects, had a significant clinical benefit from the second drug compared to the one that was taken initially^9,26–30^. These results support the potential value of developing additional novel TPO agonists, even though Romiplostim and Eltromboag have already been approved for use in humans.

### Conclusion

In conclusion, the current study reports the first evaluations of JNJ-26366821 as a radiation mitigator in mice. The results demonstrate a single dose of JNJ-26366821 provides a significant survival benefit in lethally irradiated mice, with a window of protection from 4 to 24 h post-TBI and potentially beyond. Administration of JNJ-26366821 also mitigates platelet loss and enhances the rate of platelet and neutrophil recovery in CD2F1 mice. Confirmation of efficacy was conducted in male C57BL/6 male mice as seen by the observation of trends towards increased recovery of peripheral blood cell counts at the conclusion of the 30-day study, significant increases in colony forming units and megakaryocytes in animals administered JNJ-26366821 compared to those administered saline, and recovery of FLT3-L levels in animals administered JNJ-26366821. Probit analysis of survival in animals administered JNJ-26366821 or saline revealed a dose reduction factor of 1.113 and animals exposed to lethal radiation doses showed significant increases in survival up to 6 months following irradiation and subsequent administration of JNJ-26366821 compared to animals administered saline after irradiation. Taken together, these results demonstrate increased survival and recovery with JNJ-26366821-treatment following exposure to ionizing radiation highlighting the promise of JNJ-26366821 as a radiation countermeasure.

## Materials and methods

### Animals

Male CD2F1 mice (10–11 weeks old) were purchased from Envigo (Indianapolis, Indiana) and male C57BL/6 mice (10–11 weeks old) were purchased from Jackson Laboratories (Bar Harbor, ME). The mice were housed in the Armed Forces Radiobiology Research Institute’s (AFRRI) vivarium (accredited by the Association for Assessment and Accreditation of Laboratory Animal Care-International). Experimental animals were identified by tail tattoo and housed 4 per box in sterile polycarbonate boxes with filter covers (Microisolator, Lab Products Inc., Seaford, DE) with autoclaved hardwood chip bedding. The animals received Harlan Teklad Rodent Diet 8604 and acidified water (pH 2.5–3.0) ad libitum and were acclimatized for 1–2 weeks prior to the start of each study. The animal rooms were maintained at 21 ± 2 °C and 50 ± 10% relative humidity with 10–15 cycles of fresh air hourly and a 12:12 h light:dark cycle. All procedures pertaining to animals were reviewed and approved by the AFRRI Institutional Animal Care and Use Committee (IACUC) using the principles outlined in the National Research Council’s Guide for the Care and Use of Laboratory Animals and performed in accordance with relevant guidelines and regulations. Animal studies were conducted in compliance with ARRIVE guidelines.

### Drug and drug preparation

JNJ-26366821 is a novel PEGylated TPO mimetic peptide developed by Janssen Research & Development, LLC. (Raritan, NJ). JNJ-26366821 consists of two identical 14 amino acid peptide chains of 3,295 Daltons connected by a lysine residue and linked at each N-terminal to a polyethylene glycol (PEG) chain of 20,000 Daltons. JNJ-26366821 was supplied to AFRRI in a powder form and was formulated in normal sterile saline (0.9% NaCl) before use and protected from light. Either drug or its vehicle was injected subcutaneously (SC) at the nape of the neck, post-TBI at the time indicated for each study.

### Total body irradiation (TBI) studies

Since clinical trials in which humans are exposed to lethal doses of TBI cannot ethically be conducted, the approval process for radiation countermeasures is governed by guidance issued by the FDA known as the Animal Rule^31^. Under this guidance, pre-clinical data in one or more species along with safety and dosing data in humans is used to support the approval of drugs for use in this indication. The mouse is an accepted model for development of radiation countermeasures and it was therefore used to evaluate JNJ-26366821.

Mice were irradiated bilaterally at an estimated dose rate of ~ 0.6 Gy/min in the Cobalt-60 γ-irradiation facility at AFRRI (Bethesda, MD). Animals were placed in well-ventilated plexiglass chambers made specifically for irradiating mice. An alanine/Electron Spin Resonance (ESR) dosimetry system (American Society for Testing and Material Standard E 1607) was used to measure the dose rates in the cores of acrylic phantoms (3 inches long and 1 inch in diameter) located in all empty slots of the exposure rack in the plexiglass chamber. ESR signals were measured with a calibration curve based on standard calibration dosimeters provided by the National Institute of Standard and Technology (Gaithersburg, MD). The calibration curve was verified by inter-comparison with the National Physical Laboratory in the United Kingdom. The corrections applied to the measured dose rates in phantoms were for decay of the Co-60 source and for a small difference in mass-energy absorption coefficients for water and soft tissue at the Co-60 energy level. The radiation field was previously reported to be uniform within ± 2%^19,32^.

### Housing and care of animals after irradiation

After irradiation, animals were returned to their cages and monitored three to four times daily (early morning, late morning, late afternoon and evening). Sick animals were monitored closely and a health score was given at each time of monitoring in accordance with pre-defined criteria described and approved in the IACUC protocol. A predetermined threshold health score that necessitated euthanasia was also approved in the IACUC protocol; mice that reached this threshold health score were humanely euthanized. Pain and distress were monitored using several criteria including unresponsiveness, abnormal posture, unkempt appearance, immobility, labored breathing, respiratory distress, and lack of coordination. A mouse exhibiting any of the following symptoms was determined to be moribund and was euthanized: inability of the mouse to right itself, limb paralysis, abdominal breathing, a constant twitching, trembling, or tremor that lasted for more than 10 s, or greater than 35% weight loss as per IACUC policy. Animals were euthanized according to American Veterinary Medical Association (AVMA) guidelines.

### Survival studies with JNJ-26366821 in CD2F1 and C57/BL6 mice

The survival studies in CD2F1 male mice evaluated JNJ-23666821 treatment (SC) (0.3 mg or 1 mg/kg) 24 h post-TBI and JNJ-26366821 treatment (0.3 mg/kg) 4 h post-TBI. Each treatment group for JNJ-26366821 and its vehicle (saline) contained 24 animals and the radiation dose for both studies was 9.35 Gy (~ LD70/30, 70% mortality over 30-day period). The efficacy of JNJ-26366821 as a radiation mitigator against morbidity and mortality was then further confirmed in C57BL/6 male mice. The C57BL/6 mice were administered 1 mg/kg JNJ-26366821 24 h post-TBI (8.0 Gy, ~ LD70/30) in the same manner as the CD2F1 mice. Mice in all studies were weighed prior to start of the study, animals outside ± 10% of the mean weight were excluded, and selected mice were randomized into groups of four animals per box. Survival was monitored up to four times a day when warranted for 30 days and surviving animals were euthanized at the completion of the observational period. Survival data was plotted as Kaplan–Meier plots and statistical significance of the survival differences was determined by log-rank and Fisher’s exact tests using GraphPad Prism 8 software.

### Dose and time optimization study with JNJ-26366821 in CD2F1 male mice

Five dose levels of JNJ-26366821 (0.1, 0.3, 1.0, 2.0, 3.0 mg/kg) were selected to determine the optimum single dose of JNJ-26366821 to achieve maximum efficacy at 24 h post-TBI in CD2F1 mice. Treatment and vehicle cohorts (n = 24 mice/cohort) were irradiated at 9.35 Gy (~ LD70/30) and subsequently administered one of the doses listed above or the equivalent volume of the vehicle. Another dose optimization study was conducted at 4 h post-TBI with treatment and vehicle cohorts (n = 24 mice/cohort) exposed to 9.75 Gy (~ LD 90/30). Here, 4 different doses of JNJ-26366821 (0.1, 0.3, 1.0, and 3.0 mg/kg) were tested.

After determining the optimal dose of 1.0 mg/kg in the above studies, a time optimization study was performed by administering JNJ-26366821 at different time points (4, 8 and 12 h post-TBI). Six groups, three treatment groups and three time-matched vehicle groups (n = 24 mice/group), were used in this study. Each group was irradiated at 9.75 Gy (~ LD90/30) and administered the optimal dose of JNJ-26366821 (1.0 mg/kg) or vehicle (saline) at 4, 8, or 12 h post-TBI. Animals were monitored for 30 days in the same way as described previously for all survival studies.

### Hematology studies with JNJ-26366821

To study the effects of JNJ-26366821 on recovery from hematopoietic injury following TBI, CD2F1 male mice (n = 10/group) were treated with either JNJ-26366821 (1 mg/kg) or its vehicle (saline) 24 h after TBI at a non-lethal dose of 7.0 Gy. A non-lethal dose was used to allow for animal survival while following the recovery from the hematopoietic injury that takes place following TBI. In addition, a group of sham-irradiated CD2F1 male mice were given either the drug or saline. Additionally, blood was collected from C57BL/6 male mice irradiated at an LD70/30 dose (8.0 Gy). Mice were anesthetized with isoflurane (Hospira Inc., Lake Forest, IL) and blood was collected from the submandibular vein using a 23 G needle at 2 h and 1, 3, 7, 10, 14, and 21 days post-TBI. Mice were allowed to recover fully from anesthesia and monitored closely for any signs of a post-anesthesia reaction or bleeding at the collection site before being returned to group-housed cages. Approximately 20 µL of blood was collected into tubes containing EDTA and blood was continually rotated until CBC/differential analysis of white blood cell (WBC), absolute neutrophil (NEU), monocytes (MON), lymphocyte (LYM), and platelet (PLT) counts with the HESKA Element HT™ 5 Analyzer system (HESKA Corporation, Loveland, CO).

### FLT3-L determination in serum by ELISA

Blood was collected from male C57BL/6 mice via cardiocentesis into Microtainer serum collection tubes (BD item #36596, Franklin Lakes, New Jersey) from animals anesthetized with isoflurane (5% induction, 2% maintenance); following blood collection animals were euthanized. Serum was collected following centrifugation for 10 min at 2400 × g. Circulating FLT3-L levels were determined by ELISA analysis (R&D Systems item#MFK00, Minneapolis, MN) performed according to the manufacturer’s instructions; serum was diluted 1:3.

### Colony forming unit assay from femoral bone marrow

Following euthanasia, femurs were collected and the bone marrow was extracted by flushing with IMDM with 2% FBS then plated (2 × 10^5^cells / plate) following protocols from the manufacturer (Mouse Colony-Forming Cell Assays Using MethoCult, Stem Cell Technologies, Cambridge, MA). Cultures were incubated for 14 days after plating and Granulocyte–macrophage colony forming units (CFU-GM), granulocyte-erythrocyte-monocyte-macrophage CFU (CFU-GEMM), colony-forming unit-erythroid (CFU-E) and erythroid burst-forming units (BFU-E) were identified and quantified using a Nikon TS100F microscope. Fifty or more cells were considered one colony. Data are show as mean ± standard error of mean (SEM) and statistical significance was determined between irradiated vehicle-treated and drug-treated groups^33^.

### Megakaryocytes from Sternal bone marrow

Also following euthanasia, sterna were collected on 30 days post-TBI. The sterna were fixed in a 20:1 volume of fixative (10% buffered formalin) to tissue then embedded in paraffin and longitudinal 5 μm sections were stained with regular hematoxylin and eosin (H&E) stain following standard protocol^33^. Megakaryocyte numbers were counted from each slide.

### Determination of dose reduction factor (DRF)/long term survival

Male C57BL/6 mice were distributed into one of twelve groups with 6 groups administered saline and 6 groups administered 1.0 mg/kg JNJ-26366821 24 h after TBI. The groups that ultimately received saline were irradiated at the following doses: 7.5, 8.0, 8.5, 9.0, 9.5, and 10.0 Gy. The groups that ultimately received JNJ-26366821 were irradiated at the following doses: 8.0, 8.5, 9.0, 9.5, 10.0, and 10.5 Gy. Animals were monitored as mentioned above in the methods for the survival studies. Thirty days post-TBI, the survival in each radiation dose group was used for probit analysis (IBM SPSS Statistics 25.0) to determine the LD50/30 value for both saline or JNJ-26366821treatment^34^ to calculate the DRF. Surviving animals from the DRF studies were monitored for up to 6 months post-TBI to examine long term survival.

### Statistical analysis

Survival data was plotted as Kaplan–Meier plots; GraphPad Prism 9 software was utilized to perform Fisher’s exact test to compare survival at 30 days and a log-rank test to compare survival curves. Probit analysis was conducted with IBM SPSS Statistics 25.0 and a student’s t-test was used to assess differences between groups. Averages were reported + /− standard error of the mean (SEM).

## Supplementary Information

Supplementary Information.
