# Supplementary material for: Mitigation of total body irradiation-induced mortality and hematopoietic injury of mice by a thrombopoietin mimetic (JNJ-26366821)
Source: Sci Rep. 2022 Mar 3;12:3485. doi: 10.1038/s41598-022-07426-7 (PMC8894488; doi:10.1038/s41598-022-07426-7)

Supplementary Information

Multiple JNJ-26366821 dose survival study in CD2F1 Mice

To investigate the effects of multiple doses of JNJ-26366821 administered post-TBI, CD2F1 mice were irradiated with 9.35 Gy (LD70/30 dose) and treated with 0.3 mg/kg JNJ-26366821 SC at either 24 h, 24 h + 48 h, or 24 h + 48 h + 72 h post-TBI. There were 24 animals per treatment group for JNJ-26366821 and its vehicle. The mice were monitored daily for 30 days and euthanized in moribund condition according the predetermine health score previously described.

Supplementary Figure 1: Multiple dose regimen of JNJ-26366821 (0.3 kg/kg) to improve survival of lethally irradiated CD2F1 mice when administered SC post-TBI (n=24/group). Survival curves shown here are JNJ-26366821 regimens at 0.3 mg/kg as 1 dose (●, 24 h), (■, 24 & 48 h), and (▲, 24 & 48 & 72 h) and respective vehicle controls (open symbols).

Supplementary Figure 2: Recovery of peripheral blood cells (WBCs, monocytes (MON), lymphocytes (LYM) and RBCs). Non-irradiated mice treated with saline (○) and JNJ-26366821 (●) and irradiated (7 Gy) mice treated with saline (□) and JNJ-26366821 (■). Either saline or JNJ-26366821 at 1 mg/kg was administered 24 h post-irradiation. Day 0 represents 2 h post-irradiation. Data represented are mean ± standard error of the mean (SEM) for n=10 mice. Significant difference ($p < 0.001 – 0.0125$) between JNJ-26366821 treated and saline treated irradiated groups

by ANOVA is indicated with an asterisk (*). Some data points in the figure do not have error bars that are visible because they are smaller than symbols.

Supplemental Figure 1

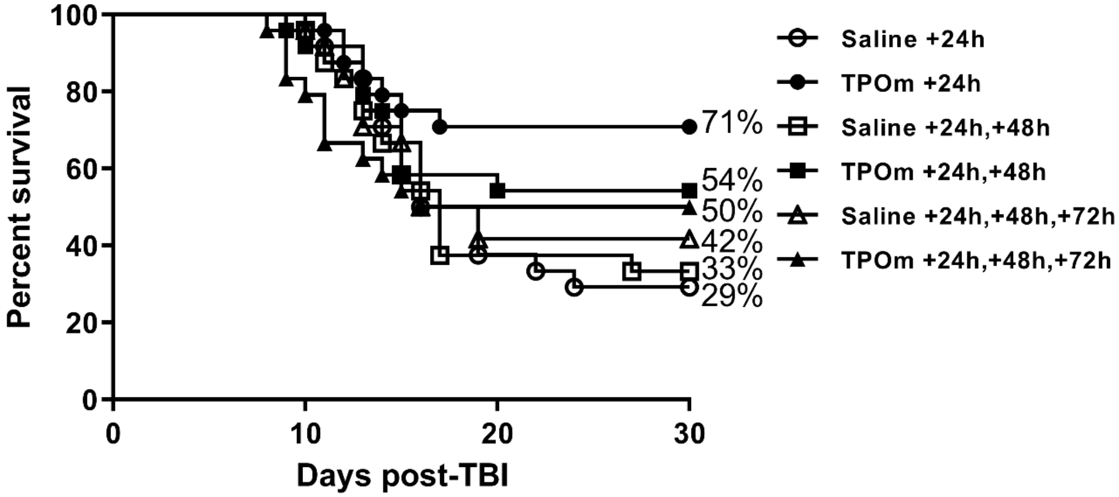

Supplemental Figure 2

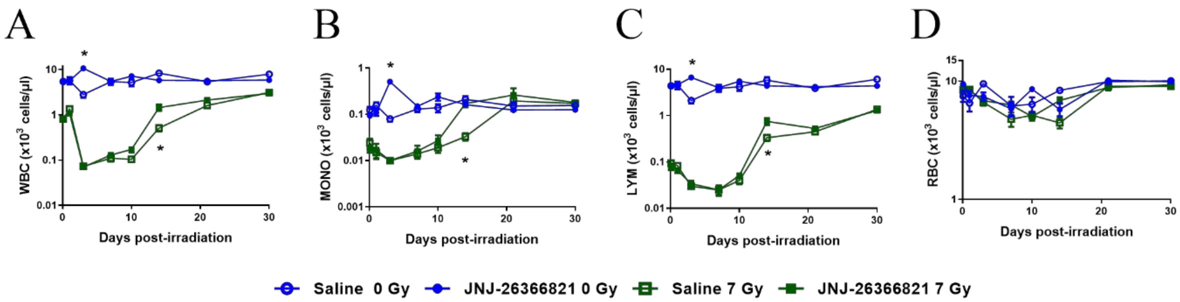

Supplement: Supplementary file 1 — Supplementary Information. [file 41598_2022_7426_MOESM1_ESM.pdf]
